# A Case of Traumatic Distal End Radius Fracture With a Hemorrhagic Volar Wrist Ganglion Cyst

**DOI:** 10.7759/cureus.61923

**Published:** 2024-06-07

**Authors:** Muhammad Firdaus, Firdaus Aslam, Rashdeen Fazwi Muhammad Nawawi

**Affiliations:** 1 Orthopaedics and Traumatology, Hospital Selayang, Selangor, MYS; 2 Orthopaedics, Hand and Microsurgery, Hospital Selayang, Kuala Lumpur, MYS

**Keywords:** ganglion cyst excision, volar wrist ganglion cyst, traumatic ganglion cyst of wrist, distal radius fracture, benign tumour

## Abstract

Wrist ganglion cysts are the most common benign soft tissue swelling in the hand and wrist. They may arise from flexor and extensor tendon sheaths, interphalangeal joints, wrist joints, and even the neural tissues around the hand and wrist. Some volar wrist ganglion cysts arise from the radiocarpal joint and scaphotrapezial joint. It is uncommonly encountered as an incidental finding during the fixation of a distal radius fracture. In our case, a volar wrist hemorrhagic ganglion cyst was incidentally found during the fixation of the fracture. Prior to the injury, the patient had no complaints of swelling over her right wrist. The cyst was removed using microscopic magnification.

## Introduction

Ganglion cysts are common soft tissue lesions that often develop in the wrist region. While most wrist ganglion cysts are considered idiopathic, there is evidence suggesting that trauma may play a role in their development. Approximately 70% of cases involve ganglion cysts arising from the dorsal aspect of the wrist, typically originating from the scapholunate joint [1]. About 20% of cysts arise from the volar wrist region near the radiocarpal joint [2]. Traumatic ganglion cysts usually result from joint or tendon sheath injuries, leading to the accumulation of synovial fluid within a cystic structure. Although most patients are asymptomatic, some may experience compressive symptoms and cosmetic dissatisfaction.

This condition rarely presents acutely; instead, it is typically diagnosed as a gradually progressive swelling over the affected site, with or without accompanying symptoms such as numbness and pain. Clinically, the mass feels firm and well-circumscribed, and it transilluminates at the affected site. Ultrasonography can help differentiate the lesion from vascular-origin swellings.

Treatment of ganglion cysts usually begins conservatively with observation, as they may resolve spontaneously. Aspiration of the ganglion cyst is an option, but it is generally avoided in volar wrist ganglion cysts due to their proximity to vascular structures. Surgical excision of the cyst is indicated primarily for symptomatic cases, but surgeons must carefully consider the lesion’s proximity to the neurovascular bundle. Volar wrist ganglion cysts have a higher recurrence rate (approximately 15% to 20%) compared to dorsal wrist ganglion cysts [3].

In our case, we encountered a volar wrist ganglion cyst during distal radius fixation. It adhered to the radial artery bifurcation near the wrist joint, making cyst excision challenging without magnification.

## Case presentation

A 58-year-old female patient was admitted to our ward after falling on her outstretched hand at home, resulting in a closed fracture of the distal end of her right radius. During the preoperative assessment, there was generalized swelling of the right wrist, bruised skin across the volar aspect of the wrist, and a limited range of motion due to pain.

**Figure 1:**
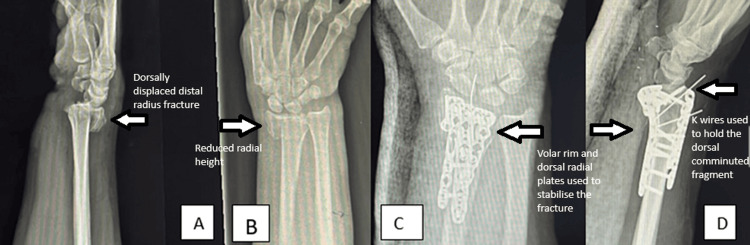
Plain radiograph of the wrist joint A, B: Plain radiograph post trauma showing AO 2R3C2 fracture; C, D: Postoperative radiograph

A plain radiograph of the wrist joint indicated significant compression of the distal radius, involving all columns and dorsal compression. The CT scan of the wrist joint revealed numerous intraarticular fragments with dorsal comminution. She was scheduled for open reduction and plating of the right radius (Figure 1)

The standard dorsal wrist approach revealed a comminuted intermediate and radial column, which were treated with two dorsal plates. During a volar approach with extended flexor carpi radialis incision, a multilobulated cystic mass was found adherent to the radial artery at the distal bifurcation of the palmar carpal branch and superficial palmar arch branch. There were numerous feeder arteries emerging from the radial artery. 

**Figure 2:**
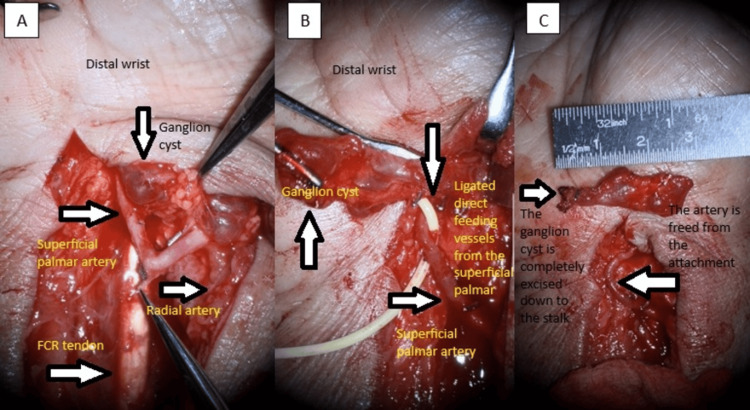
Intraoperative images A: Microscopic picture depicts a multilobulated cystic tumor near the radial artery bifurcation at the wrist; B: Ligaclip is used to ligate the radial artery's direct feeder vessels under microscope magnification; C: The hemorrhagic cyst was effectively removed from its origin (radiocarpal joint)

The cyst was meticulously detached from the artery using microsurgical equipment and microscope magnification. The feeding vessels were ligated with Ligaclip. The mass stalk was safely removed at the radiocarpal joint. A thorough inspection of the mass revealed a 3-cm multilobulated, blue mucoid substance cyst (Figure 2).

Histopathology examination of the excised mass revealed a multiloculated cystic structure with no cellular atypia, which is consistent with a ganglion cyst. Postoperatively, the patient was put on a volar slab for two weeks until the surgical wound healed. She was then instructed on wrist range of motion exercises. Three months postoperatively, she gained the full range of motion of the right wrist with no evidence of the ganglion cyst.

## Discussion

Ganglion cysts are common benign soft tissue tumors, most prevalent in women between the second and fourth decades of life [4]. Patients typically seek treatment due to cosmetic concerns and symptomatic masses, including pain, weakness, and concerns about malignant potential. Our patient did not have any mass or swelling over the injured wrist before presentation.

Theories often discussed regarding the formation of ganglion cysts include trauma, mucinous degeneration of adjacent tissue, and capsular rent [4-6]. Microscopically, the cyst wall consists of compressed collagen lining without evidence of epithelial or synovial lining, supporting the traumatic theory. The cyst contents, highly viscous mucin thicker than synovial fluid, suggest an adjacent mesenchymal tissue reaction leading to the mucinous cyst accumulation at the periarticular region, later connected to the joint with a pedicle [4]. 

Volar wrist ganglion cysts are the second most common ganglion of the hand and wrist, following dorsal wrist ganglion cysts. Common locations for ganglia include directly over the distal edge of the radius and the scaphoid tubercle. Another site for ganglion appearance is the scaphotrapezial joint. Occult dorsal wrist ganglia often occur at the scapholunate ligament region, commonly associated with chronic dorsal wrist pain, especially during wrist extension [6-8]. Treatment options for wrist ganglion cysts vary, with conservative management for asymptomatic cysts and surgical approaches including aspiration, open excision, and arthroscopic resection [9-15]. Recurrence rates after open excision may reach up to 50% [3,11].

Ganglion cysts may present directly at the joint area or be intertwined between the bifurcation of the radial artery at the wrist level. They can extend along the flexor tendon, into the carpal tunnel, and even distally into the first webspace [5]. In our case, a multilobulated cystic mass was observed at the radial artery’s distal bifurcation, involving the palmar carpal and superficial palmar arch branches. Multiple feeding channels rose directly from the radial artery.

When ganglion cysts are discovered during distal radius fracture fixation, the procedure becomes more challenging due to the potential hematoma in the surgical field, making mass excision harder. Lister et al. previously described a method for resecting a tumor adherent to the radial artery by leaving part of the tissue attached to the feeding vessels, resulting in a low recurrence rate [16]. We utilized a surgical microscope at 12.5x magnification to best visualize the cyst and surrounding structures. This guarantees that the cyst is excised securely. In light of this case, we recommend that surgeons keep at least surgical loupes readily available for similar encounters during distal radius fixation.

## Conclusions

A wrist ganglion cyst may present in an acute trauma without the patient complaining of any symptomatic mass over the wrist region. The condition may complicate fracture fixation if it is adherent to vital structures such as the radial artery in the volar wrist ganglion cyst. We may excise the mass, leaving part of the cyst capsule attached to the feeder arteries, or utilize the surgical microscope to enhance visualization of the microstructures to guarantee safe and secure excision of the mass entirely.
